# Targeted delivery of HSP90 inhibitors for efficient therapy of CD44-positive acute myeloid leukemia and solid tumor-colon cancer

**DOI:** 10.1186/s12951-024-02460-1

**Published:** 2024-04-22

**Authors:** Lejiao Jia, Huatian Yang, Yue Liu, Ying Zhou, Guosheng Li, Qian Zhou, Yan Xu, Zhiping Huang, Feng Ye, Jingjing Ye, Anchang Liu, Chunyan Ji

**Affiliations:** 1 Department of Pharmacy, Cheeloo College of Medicine, Qilu Hospital, Shandong University, Jinan, Shandong 250012 China; 2 Department of Pharmaceutics, School of Pharmaceutical Sciences, Cheeloo College of Medicine, Shandong University, Jinan, Shandong 250012 China; 3 Department of Pharmacy, Affiliated Hospital of Shandong University of Traditional Chinese Medicine (TCM), Jinan, Shandong 250014 China; 4 Department of Hematology, Cheeloo College of Medicine, Qilu Hospital, Shandong University, Jinan, Shandong 250012 China; 5 Key Laboratory of Chemical Biology of Ministry of Education, School of Pharmaceutical Sciences, Cheeloo College of Medicine, Shandong University, Jinan, Shandong 250012 China

**Keywords:** Active targeting nanoparticles, Heat shock protein 90, Acute myeloid leukemia, Solid tumor, CD44

## Abstract

Heat shock protein 90 (HSP90) is overexpressed in numerous cancers, promotes the maturation of numerous oncoproteins and facilitates cancer cell growth. Certain HSP90 inhibitors have entered clinical trials. Although less than satisfactory clinical effects or insurmountable toxicity have compelled these trials to be terminated or postponed, these results of preclinical and clinical studies demonstrated that the prospects of targeting therapeutic strategies involving HSP90 inhibitors deserve enough attention. Nanoparticulate-based drug delivery systems have been generally supposed as one of the most promising formulations especially for targeting strategies. However, so far, no active targeting nano-formulations have succeeded in clinical translation, mainly due to complicated preparation, complex formulations leading to difficult industrialization, incomplete biocompatibility or nontoxicity. In this study, HSP90 and CD44-targeted A6 peptide functionalized biomimetic nanoparticles (A6-NP) was designed and various degrees of A6-modification on nanoparticles were fabricated to evaluate targeting ability and anticancer efficiency. With no excipients, the hydrophobic HSP90 inhibitor G2111 and A6-conjugated human serum albumin could self-assemble into nanoparticles with a uniform particle size of approximately 200 nm, easy fabrication, well biocompatibility and avoidance of hepatotoxicity. Besides, G2111 encapsulated in A6-NP was only released less than 5% in 12 h, which may avoid off-target cell toxicity before entering into cancer cells. A6 peptide modification could significantly enhance uptake within a short time. Moreover, A6-NP continues to exert the broad anticancer spectrum of Hsp90 inhibitors and displays remarkable targeting ability and anticancer efficacy both in hematological malignancies and solid tumors (with colon tumors as the model cancer) both in vitro and in vivo. Overall, A6-NP, as a simple, biomimetic and active dual-targeting (CD44 and HSP90) nanomedicine, displays high potential for clinical translation.

## Introduction

Heat shock protein 90 (HSP90), known as a vital molecular chaperone, is discovered to regulate numerous cellular processes, including functional maturation, folding and activation of more than 200 client proteins [1–3]. HSP90 is overexpressed in various cancers, and is considered to promote the maturation of many oncoproteins and facilitate cancer cell growth [4, 5]. Hence, HSP90 is widely regarded as a promising target for the treatment of numerous human cancers [6–10]. Since 1999, when tanespimycin (17-AAG) was introduced as the first-in-class Hsp90 inhibitor to enter clinical trials, over one hundred Hsp90 inhibitor oncology trials have been conducted and the broad spectrum of cancer (solid and hematological cancers) is the most common indication [1, 10–12]. Unfortunately, most candidates are deemed clinical failures owing to toxicity, low efficacy, or drug resistance. Although the number of clinical trials on Hsp90 inhibitor monotherapy is decreasing, the proportion of Hsp90 inhibitors combined with targeted agents achieving much stronger anticancer efficacy has been an increase, and the prospects of their application deserve our enough attention [7, 13–16].

Over the past several decades, nanoparticulate-based drug delivery systems have been extensively investigated and generally supposed as one of the most promising formulations especially for targeted delivery, reduced toxicity of anticancer drugs and improved efficacy [17–19]. However, despite progress and promise, so far, no active targeting nano-formulations have succeeded in clinical translation, mainly due to complicated process control, complex formulations leading to difficult industrialization, incomplete biocompatibility or nontoxicity [20, 21]. The balance among functionality, simplicity and biocompatibility is key to the success of targeted nano-formulations [22]. An antibody-drug conjugate (ADC) or a peptide-drug conjugate (PDC) that is conjugated an antibody or a peptide to a drug via a linker is a promising therapeutic modality with simplicity and biocompatibility, and has created opportunities for some candidates with poor druggability to enter clinical treatments, such as maytansine [23–26]. Compared with that of an antibody, the size of a peptide is much smaller. Antibodies (IgG) typically consist of more than 1000 amino acids (∼ 150 kDa), while peptides employed for cancer cell targeting range in length from 5 to 25 amino acids [27]. Therefore, peptides are easy to scale up, modify and have good immunogenicity in the body [28, 29]. Although linkers in peptide-drug conjugates require specific environmental conditions (temperature, pH, redox conditions, etc.) to break off and release the drug, with the drug sometimes not being released as a prototype drug or even not being released [30], it gave us the inspiration to conjugate peptides to biomacromolecules via linkers and drugs could be loaded into biomacromolecules, which can release payloads with them metabolized in physiological environments. In our study, human serum albumin (HSA) was chosen as the biomacromolecule conjugated targeting peptide via a linker. HSA, the most abundant protein in plasma, has been regarded as a prominent anticancer drug carrier owing to its well biocompatibility, long half-life, ability to transport a variety of endogenous and exogenous cargoes [31]. Besides, nutrient-deficient cancer cells actively take up and metabolize albumin to provide amino acids and energy needed for rapid proliferation.

**Fig. 1:**
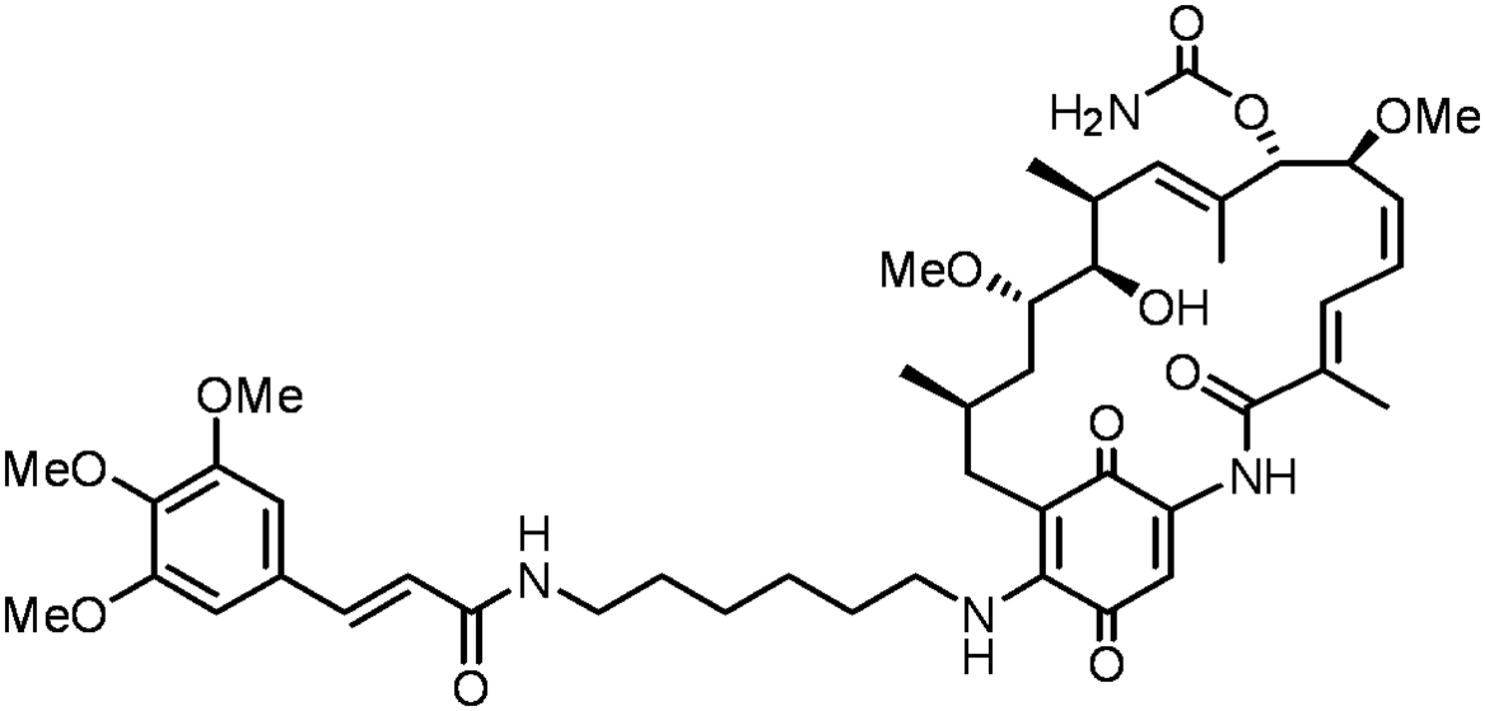
The chemical structure of G2111

**Scheme 1:**
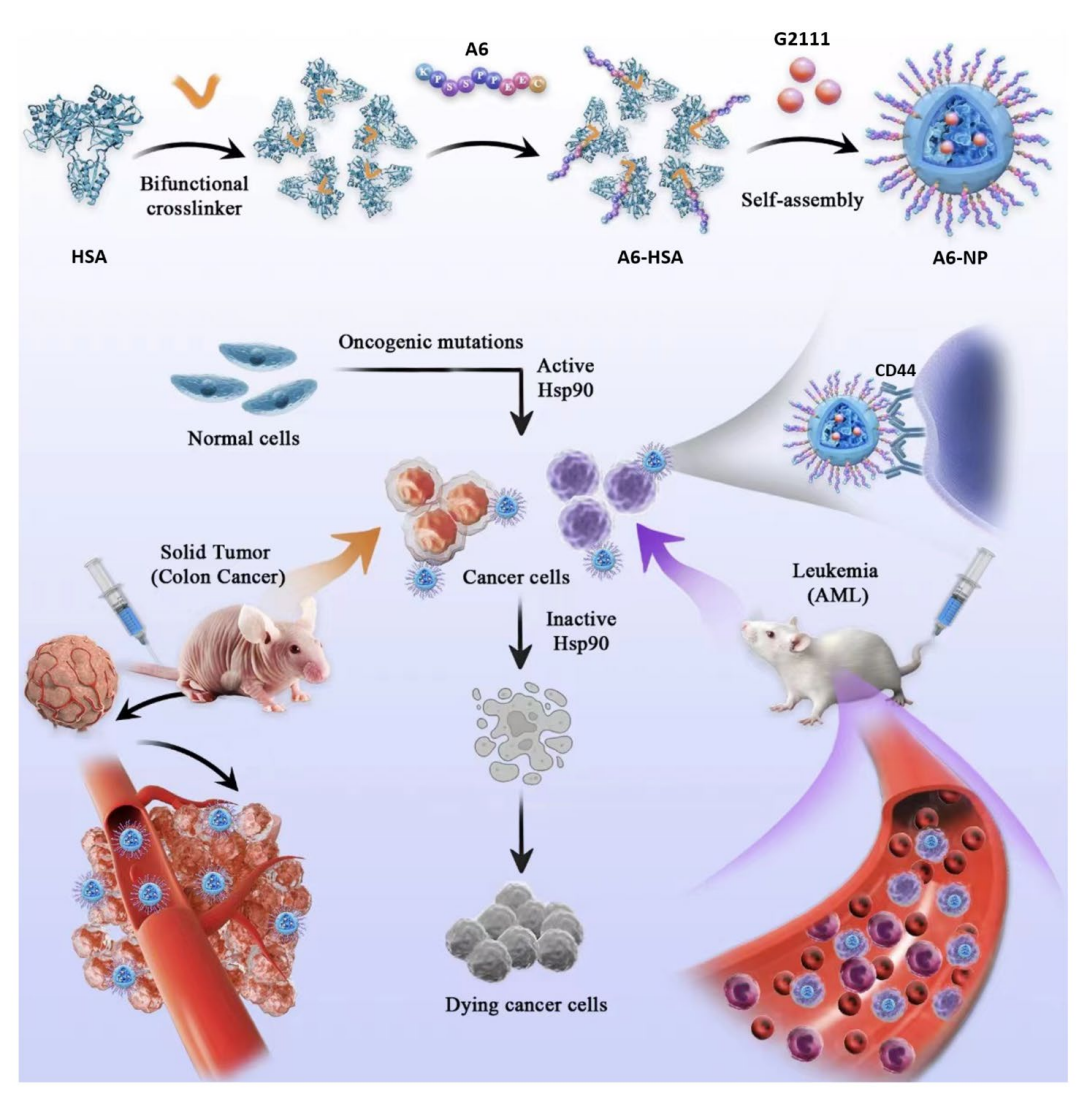
Schematic illustration on the fabrication of A6-NP and target therapy of acute myeloid leukemia and solid tumor-colon cancer

To continue to exert the broad anticancer spectrum of Hsp90 inhibitors, the target peptide we selected is best also highly expressed both in solid tumors and hematological malignancies. As a non-kinase cell surface transmembrane glycoprotein, CD44 is highly expressed in various human cancer cells [32]. Moreover, for a large number of solid tumors and leukemic cancers, CD44-targeted nanomedicines have been reported to be promising treatments [33–35]. A6, a urokinase-derived peptide with eight L-amino acids (Ac-KPSSPPEE-NH_2_), has been proven to specially target and bind to CD44 [36]. Notably, the A6 peptide has been reported to exhibit an excellent safety profile in animal studies and phase I and II clinical trials [37]. Besides, our group has been committed to the development and synthesis of Hsp90 inhibitors [38], among which the candidate compound (the structure is shown as Fig. 1, named G2111 in this paper) showed higher activity than 17AAG but lower liver toxicity. Therefore, in this study, CD44-targeted A6 peptide conjugated HSA (A6-HSA) was first synthesized and G2111 was loaded into HSA to form A6-HSA-based nanoparticles (A6-NP) through self-assembly via a convenient ultrasound method. Our fabricated A6 peptide functionalized nanoparticles with CD44 and Hsp90 targets are expected to possess a broad anti-cancer spectrum both in hematological malignancies and solid tumors, which are rarely reported in nanomedicines. Hence, biocompatibility, targetability and anticancer activity in vitro and in vivo were systematically investigated in acute myeloid leukemia and solid tumor-colon cancer models in the present study. The main scheme is presented in Scheme 1.

## Materials and methods

### Materials and reagents

G2111 was synthesized in our laboratory. Human Serum Albumin (HSA) (20%) was purchased from CSL Behring AG (Philadelphia, PA, USA). N-Succinimidyl 4-(maleimidomethyl) cyclohexane-1-carboxylate (SMCC) was obtained from Beijing Innochem Science & Technology Co., Ltd (Beijing, China). A6 peptide was provided by GUOTAI Biotechnology (Beijing, China). Coumarin 6 and IR780 were purchased from Sigma-Aldrich Co. (St Louis, MO, USA). Antibodies against CDK4 and β-actin were obtained from Abcam (Cambridge, MA, USA). Cell Counting Kit-8 (CCK-8) was from Shanghai Epizyme Biomedical Technology Co., Ltd (Shanghai, China). The commercial assay kit for Annexin V-FITC apoptosis detection was acquired from Bestbio (Shanghai, China). Aspartate aminotransferase (AST) assay kit and Alanine aminotransferase (ALT) assay kit were obtained from Nanjing Jiancheng Bioengineering Institute (Nanjing, China).

### Cell culture and animals

The human acute myeloid leukemia cell line MOLM13 and the human colon cancer cell line HCT116 were cultivated in RPMI-1640 medium (Gibco) supplemented with 10% FBS (Gibco) and 100 U/mL of penicillin and 100 mg/mL of streptomycin (Gibco). The human umbilical vein endothelial cell line (HUVEC) was cultured in Dulbecco’s modified Eagle’s medium (DMEM, Gibco) supplemented with 10% FBS and 1% penicillin/streptomycin. All the cells were placed in an incubator with 5% CO_2_ at 37 °C.

NOD-Prkdc^scid^-Il2rg^em1IDMO^ (NPI) mice (male, 6 ∼ 8-weeks old) were obtained from the Beijing IDMO CO.,Ltd. Orthotopic AML murine model was established via transplanting 1 × 10^6^ MOLM-13 cells. Balb/c nude mice (female, 18 ∼ 22 g) were purchased from Jinan Pengyue Experimental Animal Breeding Co., Ltd (Jinan, China). The murine model of heterotopic colon cancer was established by injecting 100 µL of HCT116 cells (2 × 10^6^) containing 50% Matrigel into the right axilla of each mouse. Animal protocols were authorized by the Animal Ethics Committee of Qilu Hospital of Shandong University.

### Fabrication and characterization of A6-NP

#### Synthesis of A6-HSA

To construct A6 peptide-functionalized HSA, SMCC was chosen as a bifunctional linker between A6 peptide and HSA. First, SMCC and A6 peptide were dissolved in DMSO (5 mg/mL) and PBS (6 mg/mL) respectively. Different levels of SMCC were added to 4 mL of HSA solution dropwise under stirring at room temperature for 30 min, and then A6 solution was added and stirred for another 30 min. After being washed with PBS for 3 times by the ultrafiltration method, the modified HSA was obtained and then lyophilized.

#### Coomassie brilliant blue staining and MALDI-TOF-MS measurements

To detect whether the A6 peptide was successfully linked to HSA, coomassie brilliant blue staining was used to qualitatively determine the difference in the molecular weight of HSA after modification. SDS-PAGE was performed using a Bio-Rad vertical electrophoresis system at 80 V for 30 min, which was subsequently changed to 120 V for 50 min. After electrophoresis, the SDS-PAGE gel was washed 3 times with double-distilled water to remove the residual SDS from the gel surface. Then the gel was incubated with coomassie blue fast staining solution for 2 h at room temperature. Subsequently, bright and clear bands were obtained after being washed with double-distilled water to remove the background color.

Matrix-assisted laser desorption/ionization time-of-flight mass spectrometry (MALDI-TOF-MS) was used to quantitatively examine the modification of A6 peptide linked to HSA through SMCC. The freeze-dried samples were re-dissolved in TA50 (0.1% trifluoroacetic acid (TFA): acetonitrile = 1:1). The mass spectra of HSA modified by different degrees of A6 peptide was acquired in a mass range between 30,000 Da and 200,000 Da.

#### Fabrication of A6-HSA-based nanoparticles (A6-NP)

The Hsp90 inhibitor G2111 was dissolved in dichloromethane at the concentration of 10 mg/mL. Then, 200 µL of G2111 was added to 1 mL of modified HSA followed by ultrasonication using a probe horn, and the temperature was controlled in an ice bath. After ultrasonication, the mixture was immediately stirred at 600 rpm at 40 °C to remove the dichloromethane. Centrifugation and passing through a sterile 0.22 μm microporous filter membrane were performed to obtain the A6-NP. The preparation of nanoparticles without A6 peptide modification was the same as above except for the use of HSA instead of A6-HSA.

#### Drug loading determination

To quantify the efficiency of drug loading, G2111 was analyzed by HPLC (Agilent, Santa Clara, CA, USA). 50 µL of sample was vortex-mixed with 200 µL of methanol for 2 min to disturb structures of nanoparticles, and resultant samples were centrifuged for 20 min (14,000 rpm). The HPLC assay was applied to analyze the supernatant. The mobile phase was composed of 25% (v/v) sodium phosphate buffer (25 mM, pH = 3) with 10 mM triethylamine and 75% methanol at a flow rate of 1.0 mL/min. The detection wavelength of G2111 was set at 230 nm. The protein content in nanoparticles was determined with a BCA assay kit. The drug loading content was calculated as follows: Drug loading efficiency (%) = Mass of drugs in nanoparticles/Total mass of nanoparticles×100%.

#### Characterization of A6-NP

The size and ζ-potential analysis of fabricated nanoparticles were performed by Zetasizer Nano-ZS + MPT-2 (Malvern Instruments Ltd, UK). Transmission Electron Microscope (TEM) JEM-1200EX II (JEOL Co., Ltd, Japan) was carried out to detect the morphology of samples. X-RD patterns of the samples were determined by an X-ray diffractometer (SmartLab 9KW, Rigaku, Japan). Diffractograms were displayed from 3° to 50°(2*θ*), at a scanning speed of 4°/min.

### In vitro drug release assay

To determine the drug release behavior, different nanoparticle suspensions were investigated by the dialysis method. Briefly, 0.5 mL of NP, 2.3%A6-NP or 5.5%A6-NP was thrown into dialysis bags (MWCO 8000 − 14,000 Da), and then these sealed dialysis bags were immersed into the release medium with a total volume of 20 mL. Subsequently, the system was performed in a 37 °C air bath shaker (100 rpm). At different time points, 1 mL of sample was withdrawn and centrifuged at 14,000 rpm for 20 min for analysis by an HPLC system, and equal volumes of fresh release medium were immediately added.

### Cellular uptake of A6-NP

To determine the internalization of nanoparticles into the acute myeloid leukemia cell MOLM13 and the human colon cancer cell HCT116, coumarin 6 was used as a fluorescent probe to label nanoparticles. At 80 − 90% confluence, HCT116 cells were treated with a series of nanoparticles for 0.5 h. MOLM-13 cells were seeded in 12-well plates at a density of 5 × 10^5^ cells/well and incubated with nanoparticles for 0.5 h. HCT116 cells or MOLM-13 cells were washed three times with PBS and photographed by fluorescence microscopy. Quantitative study on cellular uptake of nanoparticles was analyzed by flow cytometry (Novocyte, Agilent).

### In vitro evaluation of cytotoxicity and hemolysis studies

The cytotoxicity of blank nanoparticles was determined by Cell Counting Kit-8 assay. Briefly, HCT116 cells, MOLM13 cells and HUVECs were separately seeded in 96-well dishes for 24 h at densities of 4 × 10^3^/well, 2 × 10^4^/well and 4 × 10^3^/well, respectively, and then treated with different concentrations of blank nanoparticles for another 48 h. Afterward, 10 µL of CCK-8 was added to each well, followed by incubation for another 3 h. The absorbance intensity at 450 nm for each well was detected.

Hemolysis studies were conducted to investigate the safety of our fabricated nanoparticles. NP, 2.3%A6-NP and 5.5%A6-NP were added to 2% red blood cell suspensions at different concentrations. For comparison, the same volumes of normal saline and double distilled water were regarded as the negative and positive controls, respectively. After incubating for 3 h at 37 °C, the samples were centrifuged for 10 min at 3000 rpm. The absorbance of the supernatant at 541 nm was determined. The hemolysis percentage (%) = [(A_sample_-A_negative control_)/ (A_positive control_-A_negative control_)] ×100%.

### Cell apoptosis

MOLM13 cells were cultured in a 12-well plate (4 × 10^5^ per well) with free G2111, NP, 2.3%A6-NP and 5.5%A6-NP incubation for 24 h. Then, cells were washed with cold PBS and apoptotic cells were analyzed by an annexin V-FITC/PI detection kit and assayed by flow cytometry.

### In vitro anticancer efficiency of A6-NP

The in vitro anticancer effect of Hsp90 inhibitor-loaded nanoparticles was studied in HCT116 and MOLM-13 cells. Briefly, HCT116 and MOLM13 cells were separately cultured in 96-well plates for 24 h at a density of 4 × 10^3^ per well and 2 × 10^4^ per well. Then, different concentrations of G2111, NP, 2.3%A6-NP and 5.5%A6-NP were added. After 48 h incubation, 10 µL of CCK8 was added to each well for another 3 h, after which the absorbance at 450 nm was measured.

### Western blot analysis

HCT116 cells and MOLM13 cells were seeded in six-well plates and then incubated with NP, 2.3%A6-NP and 5.5%A6-NP, respectively. After 48 h, cell samples were collected for protein sample preparation. The protein concentration was detected using BCA protein assay kit. Then, the samples were separated by SDS-PAGE gels and electro-transferred onto polyvinylidene fluoride (PVDF) membranes. After blocking, the membranes were incubated in specific primary antibody overnight at 4 °C and secondary antibody for 1 h at room temperature. The results were analyzed using Lane 1D software.

### In vivo antileukemic capability of A6-NP

Establishment of the orthotopic AML mice model was conducted by transplanting 1 × 10^6^ of MOLM-13 cells into NPI mice via the tail vein injection. The orthotopic AML mice were divided into 5 groups (*n* = 5), and NS (normal saline), free G2111, NP, 2.3%A6-NP or 5.5%A6-NP (G2111 5 mg kg^− 1^) was administered on the third day after transplantation. Then, different treatments were conducted every other day for a total of five doses and the body weight of mice was recorded during the treatment period. Peripheral blood was collected from eye sockets on day 9. WBC counts, PLT counts and HGB level of peripheral blood were measured using an auto-hematology analyzer. The changes in ALT and AST levels in serum were detected by aspartate aminotransferase (AST) assay kit and alanine aminotransferase (ALT) assay kit, respectively. The survival was monitored through the Kaplan-Meier survival plot presented.

### In vivo leukemia cell infiltration and histological analyses

To study the influence of anticancer therapy on leukemia cell invasion and its effect on the spleen and bone marrow, orthotopic AML mice, which were administered five injections of NS (normal saline), free G2111, NP, 2.3%A6-NP or 5.5%A6-NP (G2111 5 mg kg^− 1^) every other day on days 0–8, were dissected on day 9. The organs, peripheral blood and bone marrow were collected for evaluation. The cells collected from peripheral blood, spleen and bone marrow were resuspended in PBS, and then treated with red blood cell lysis buffer solution for 5 min to lyse red blood cells. Then, these obtained cells were stained with anti-FITC-CD45 antibody for 20 min and quantified using flow cytometry. The infiltration of leukemia cells in the spleen and bone marrow was further assessed by H&E staining which performed as per standard protocols. Meanwhile, histological analyses for the heart, liver, lung and kidney were conducted.

### In vivo biodistribution of A6-NP in HCT116 colon tumor-bearing mice

Real-time fluorescence imaging in vivo was performed to assess active targeting and tumor accumulation of A6-NP. G2111 was replaced by IR780 to form formulations for imaging. The in vivo biodistribution of A6-NPs was evaluated in a HCT116 tumor-bearing mouse model. When the solid tumors reached a volume of 250 mm^3^, the mice were randomly divided into 4 groups. 0.1 mL of free IR780, NP, 2.3%A6-NP or 5.5%A6-NP was injected via tail veins. At the predetermined time, the fluorescence intensity was detected through the IVIS® Kinetic Imaging system. After 24 h, the mice were euthanized and the tumors and main tissues were harvested for ex vivo imaging.

### In vivo antitumor efficacy of A6-NP against HCT116 colon tumors

The in vivo therapeutic efficacy and toxicity were assessed in HCT116 tumor xenograft bearing BALB/c mice which was established by injecting 100 µL of HCT116 cells (2 × 10^6^) containing 50% Matrigel into the right axilla of each mouse. When the tumor volume reached 80–100 mm^3^, the mice were randomly divided into 5 groups (*n* = 5), namely, NS, G2111, NP, 2.3%A6-NP and 5.5%A6-NP group. The mice were intravenously injected with free G2111 or NPs via vein every other day for 5 times (G2111 5 mg kg^− 1^). The therapeutic efficacy and safety were estimated by measuring tumor volumes and monitoring body weights. Tumor volume was calculated as follows: V = (Length) × (Width)^2^/2.

On day 11, the mice were sacrificed. The tumors, major organs (heart, liver, spleen, lung and kidney) were dissected and fixed in 4% formaldehyde for paraffin embedding and sections. Hematoxylin and eosin (H&E) staining was performed as per standard protocols. The tumor sections were further stained with Ki67 or TUNEL for evaluation of cell proliferation or tissues apoptosis.

## Results and discussion

### Synthesis of A6-HSA

**Fig. 2:**
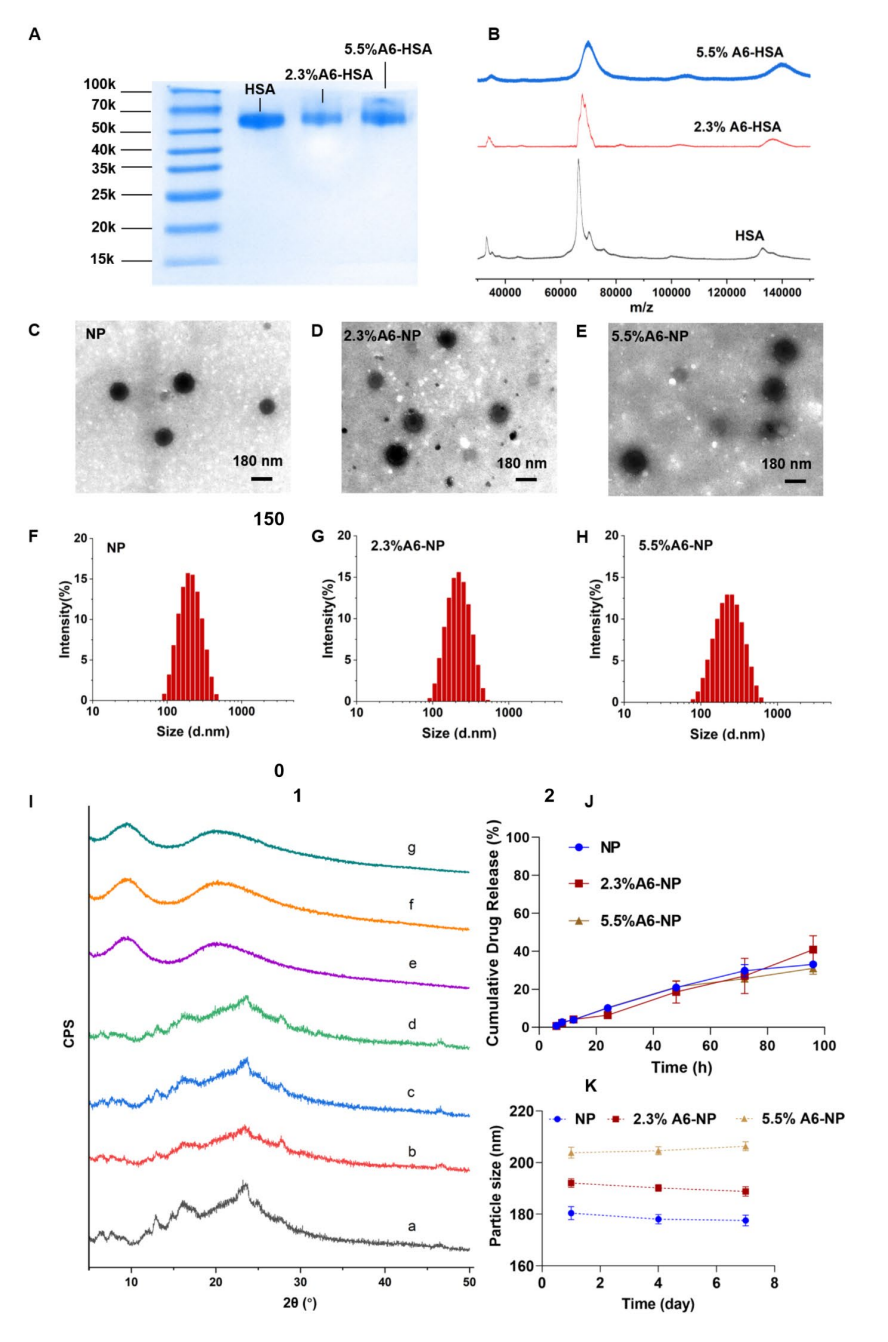
Synthesis and characterization of A6-NP. (**A**) SDS-PAGE of HSA, 2.3%A6-HSA and 5.5%A6-HSA, respectively. (**B**) MADLI-TOF-MS of HSA, 2.3%A6-HSA and 5.5%A6-HSA, respectively. (**C-E**) TEM image of NP, 2.3%A6-NP and 5.5%A6-NP, respectively. (**F-H**) Hydrodynamic size of NP, 2.3%A6-NP and 5.5%A6-NP, respectively. (**I**) X-ray diffraction patterns: **a** G2111, **b** the physical mixture of blank-NP and G2111, **c** the physical mixture of blank-2.3%A6-NP and G2111, **d** the physical mixture of blank-5.5%A6-NP and G2111, **e** NP, **f** 2.3%A6-NP, **g** 5.5%A6-NP. (**J**) In vitro drug release curves (*n* = 3). (**K**) The stability of nanoparticles within 7 days at 4 °C

Inspired by an antibody-drug conjugate or a peptide-drug conjugate, N-succinimidyl 4-(maleimidomethyl) cyclohexane-1-carboxylate (SMCC) was chosen as the biofunctional crosslinker between A6 and human serum albumin (HSA), which has been applied in some ADCs ongoing clinical trials or on the market, and has proven to be well biocompatible [39, 40]. Therefore, A6 peptide was conjugated to HSA by SMCC through strong covalent bonding, which could be stable during circulation to avoid off-target effects. Once nanoparticles are internalized into cancer cells, albumin can be metabolized to release G2111. The A6 peptide-modified HSA was synthesized by a convenient and mild coupling reaction. Various degrees of HSA modification by the A6 peptide were obtained to further evaluate the targeting ability and anticancer efficiency. Characterization of A6 peptide-conjugated HSA (A6-HSA) was performed using SDS-PAGE Coomassie blue staining and MADLI-TOF-MS, both qualitatively and quantitatively. As shown in Fig. 2A, the results revealed a decreasing electrophoretic migration distance between HSA and A6-HSA. The differences in molecular weight indicated the successful conjugation of A6 peptide to HSA through SMCC. The matrix-assisted laser desorption/ionization time-of-flight mass spectrometry (MADLI-TOF-MS) was further applied to analyze the obtained peptide-conjugated albumin [41]. The results revealed that the molecular-related ion peaks of HSA and different degrees of A6-HSA were at m/z 66,464, 67,993 and 70,119, respectively (Fig. 2B).

In our construction of peptide-linked albumin, the A6 peptide must be linked to albumin through SMCC. Therefore, at the beginning of feeding, we controlled the molar ratios of SMCC to albumin to 1:1 and 3:1, respectively. After SMCC was coupled with albumin, excess A6 peptide was added to react with SMCC, and the unreacted A6 peptide was removed by ultrafiltration. Therefore, the MADLI-TOF-MS results combined with the feeding ratio confirmed the successful conjugation of the A6 peptide to human serum albumin. Because A6 is linked to albumin via SMCC, A6 and SMCC were considered as a whole to calculate the degree of modification. The modification degree (MD) was calculated using the following equation and the degrees of coupling were calculated to be 2.3% and 5.5% (m/m), respectively.

$$\eqalign{{\rm{MD}}\% {\mkern 1mu} & \cr & {\rm{ = }}{\mkern 1mu} {{{\rm{the}}{\mkern 1mu} {\rm{molecularweight}}{\mkern 1mu} {\rm{of}}{\mkern 1mu} {\rm{product}}{\mkern 1mu} {\rm{ - }}{\mkern 1mu} {\rm{the}}{\mkern 1mu} {\rm{molecular}}{\mkern 1mu} {\rm{weight}}{\mkern 1mu} {\rm{of}}{\mkern 1mu} {\rm{HSA}}} \over {{\rm{the}}{\mkern 1mu} {\rm{molecular}}{\mkern 1mu} {\rm{weight}}{\mkern 1mu} {\rm{of}}{\mkern 1mu} {\rm{HSA}}}}{\mkern 1mu} \cr & \times {\mkern 1mu} {\rm{100}}\% \cr}$$

### Fabrication and characterization of A6-HSA-based nanoparticles

CD44-targeted A6-HSA-based nanoparticles were fabricated through driving hydrophobic G2111 and A6-HSA self-assembling without any excipients via ultrafiltration, which was simple, low-cost and time-saving. The morphology of NP, 2.3%A6-NP and 5.5%A6-NP were observed by transmission electron microscopy (TEM). As shown in Fig. 2C-E, all the nanoparticles were disseminated as individual particles with a well-defined spherical structure, and the particle size ranged from approximately 200 nm. The NP was a particle with a smooth surface (Fig. 2C), however, the surface of the nanoparticles functionalized with A6 peptide was blurred and the boundaries were not as defined as those of NP probably due to the surface conjugated with the A6 peptide (Fig. 2D-E). Besides, the greater the degree of modification of A6 was, the fuzzier the surface of A6-NP exhibited. The particle size, size distribution and ζ-potential were measured using dynamic light scattering. As shown in Fig. 2F; Table 1, the average hydrodynamic size of G2111 loaded NP was approximately 180.4 nm, while the size of 2.3%A6-NP was slightly increased. When nanoparticles were constructed with 5.5%A6-HSA, the particle size further increased (203.8 nm), which confirmed that A6 peptide was conjugated on the surface of nanoparticles. Besides, NP, 2.3%A6-NP and 5.5%A6-NP all exhibited negative surface charges of − 11.9, − 11.2, and − 11.7 mV, respectively. These results showed that A6-NPs were successfully fabricated.

**Table 1:** Characterization parameters of nanoparticles (*n* = 3)

Sample	Particle size (nm)	Polydispersity index	Zeta (mV)	Drug loading (%)
NP	180.4 ± 2.5	0.149 ± 0.030	-11.9 ± 0.7	4.59 ± 0.52
2.3%A6-NP	192.1 ± 1.7	0.173 ± 0.010	-11.2 ± 1.0	3.74 ± 0.48
5.5%A6-NP	203.8 ± 2.1	0.180 ± 0.017	-11.7 ± 0.95	3.95 ± 0.20

To explore whether the crystalline state of G2111 changed after it was loaded into our prepared nanoparticles, X-ray diffraction (XRD) patterns of the samples were performed. As Fig. 2I demonstrated, pure G2111 exhibited noticeable characteristic crystalline diffraction peaks at 2*θ* equals 13.1º, 16.2º and 23.8º. Additionally, the characteristic diffraction peaks of the physical mixture of NP, 2.3%A6-NP, 5.5%A6-NP and G2111 were observed. However, unlike those of the physical mixture, characteristic diffraction peaks could not be found for the drug-loaded NP, 2.3%A6-NP or 5.5%A6-NP (Fig. 2I). In addition, the XRD patterns of drug-loaded NP, 2.3%A6-NP and 5.5%A6-NP exhibited broad and weak diffusion peaks. The absence of characteristic peaks suggested that G2111 loaded in nanoparticles were in a non-crystalline state. The efficiency of G2111 loading was analyzed by HPLC, and the drug loading efficiencies of NP, 2.3%A6-NP and 5.5%A6-NP were 4.59 ± 0.52, 3.74 *±* 0.48 and 3.95 *±* 0.20, respectively (Table 1).

The results of stability tests are shown in Fig. 2K. Initially, hydrophobic G2111 and A6-HSA could self-assemble into nanoparticles with a uniform size distribution, and the hydrodynamic diameters of NP, 2.3%A6-NP and 5.5%A6-NP were 180.4 ± 2.5 nm, 192.1 ± 1.7 nm and 203.8 ± 2.1 nm, respectively. Moreover, the particle sizes of the three kinds of nanoparticles remained almost unchanged for 7 days at 4 °C, suggesting the favorable colloidal stability of A6-NPs.

### In vitro drug release assay

The in vitro drug release behaviors of our fabricated nanoparticles were studied in PBS (pH = 7.4, containing 0.5% Tween 80). The cumulative drug release curves are shown in Fig. 2J. The G2111 from NP, 2.3%A6-NP and 5.5%A6-NP was released slowly and the release percentage of all nanoparticles could not reach to 10% at 24 h. In the follow-up experiment, the drugs in NP, 2.3%A6-NP and 5.5%A6-NP continued to release slowly, and the cumulative drug release rates were only 33.6%, 40.9% and 31.0%, respectively, even at 96 h. We synthesized A6-HSA using the linker SMCC and then encapsulated G2111 into A6-HSA-based nanoparticles. Rather than the conjugation of A6 peptide to the surface of nanoparticles, the latter probably causes drug leakage and loss in blood circulation before entering cancer cells. Then, the in vitro release of G2111 from A6-NP was estimated under simulated physiological conditions. The above results revealed that G2111 encapsulated in nanoparticles were only released less than 5% in 12 h. Therefore, most drugs are in nanoparticles, which can be transported more to cancer cells by the A6 targets and reduce off-target cell toxicity. After nanoparticles are taken up by cancer cells, the framework of the nanoparticles is broken up due to the metabolism of albumin by cells, and drugs can be released to exert antitumor effects.

### Cellular uptake in vitro

**Fig. 3:**
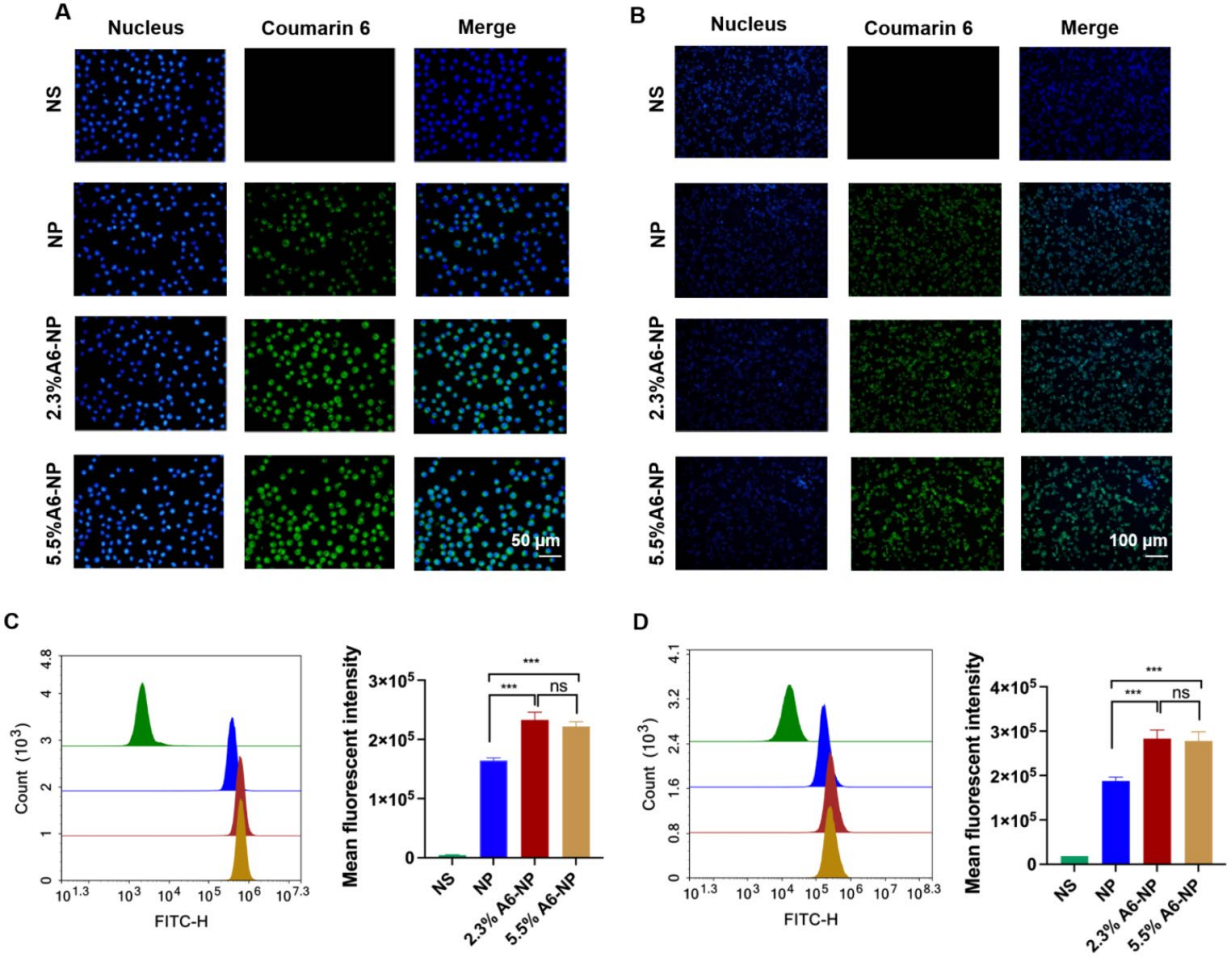
In vitro cellular uptake. (**A**) Fluorescence images of MOLM-13 cells incubated with different nanoparticles for 0.5 h; green represents C6 and blue represents nucleus. (**B**) Fluorescence images of HCT116 cells incubated with different nanoparticles for 0.5 h; green represents C6 and blue represents nucleus. (**C**) Flow cytometric analyses of MOLM-13 cells incubated with nanoparticles (*n* = 3). (**D**) Flow cytometric analyses of HCT116 cells incubated with nanoparticles (*n* = 3). ^***^p < 0.001

A6 peptide, as a CD44-targeting molecule, was conjugated to the surface of albumin-based nanoparticles. To investigate whether the A6 peptide modification could enhance the targeting of CD44^+^ acute myeloid leukemia MOLM13 cells and solid tumor-Colon cancer HCT116 cells, Coumarin 6-labeled nanoparticles with varying A6 peptide contents were applied to study the cellular uptake characteristics of nanoparticles. Qualitative and quantitative analyses were performed via fluorescence imaging and flow cytometry, respectively. As exhibited in Fig. 3A, very little green fluorescence was observed when MOLM13 cells were treated with NP for 0.5 h, implying poor uptake of unmodified A6 nanoparticles in a short time. However, the fluorescence intensity of leukemia cells treated with the A6-NP was very strong for only 0.5 h, demonstrating that nanoparticles modified with A6 peptide could enter leukemia cells within a short time more quickly. According to the results of the quantitative flow cytometry analysis, A6-NP also exhibited significantly greater cellular uptake into MOLM13 cells compared with NP (Fig. 3C, *p* < 0.001). There was no significant difference between the fluorescence intensity of 2.3%A6-NP and that of 5.5%A6-NP group, although the degree of coupling with A6 peptide was almost twice that of 2.3%A6-NP.

The characteristics of leukemia are very different from those of solid tumors, hence, the internalization of NP, 2.3%A6-NP or 5.5%A6-NP into solid tumor cells was assessed in solid tumors with colon cancer HCT116 cells as model cells. The fluorescence intensities in HCT116 cells treated with 2.3%A6-NP and 5.5%A6-NP for 0.5 h were significantly higher than those in cells treated with NP (*p* < 0.001), indicating that NP modified with A6 peptide could also be quickly and easily taken up by colon cancer cells (Fig. 3B and D). Compared with that in the 2.3%A6-NP group, the fluorescence in the 5.5%A6-NP group was not markedly difference.

### Hemolysis assay and cytotoxicity evaluation

**Fig. 4:**
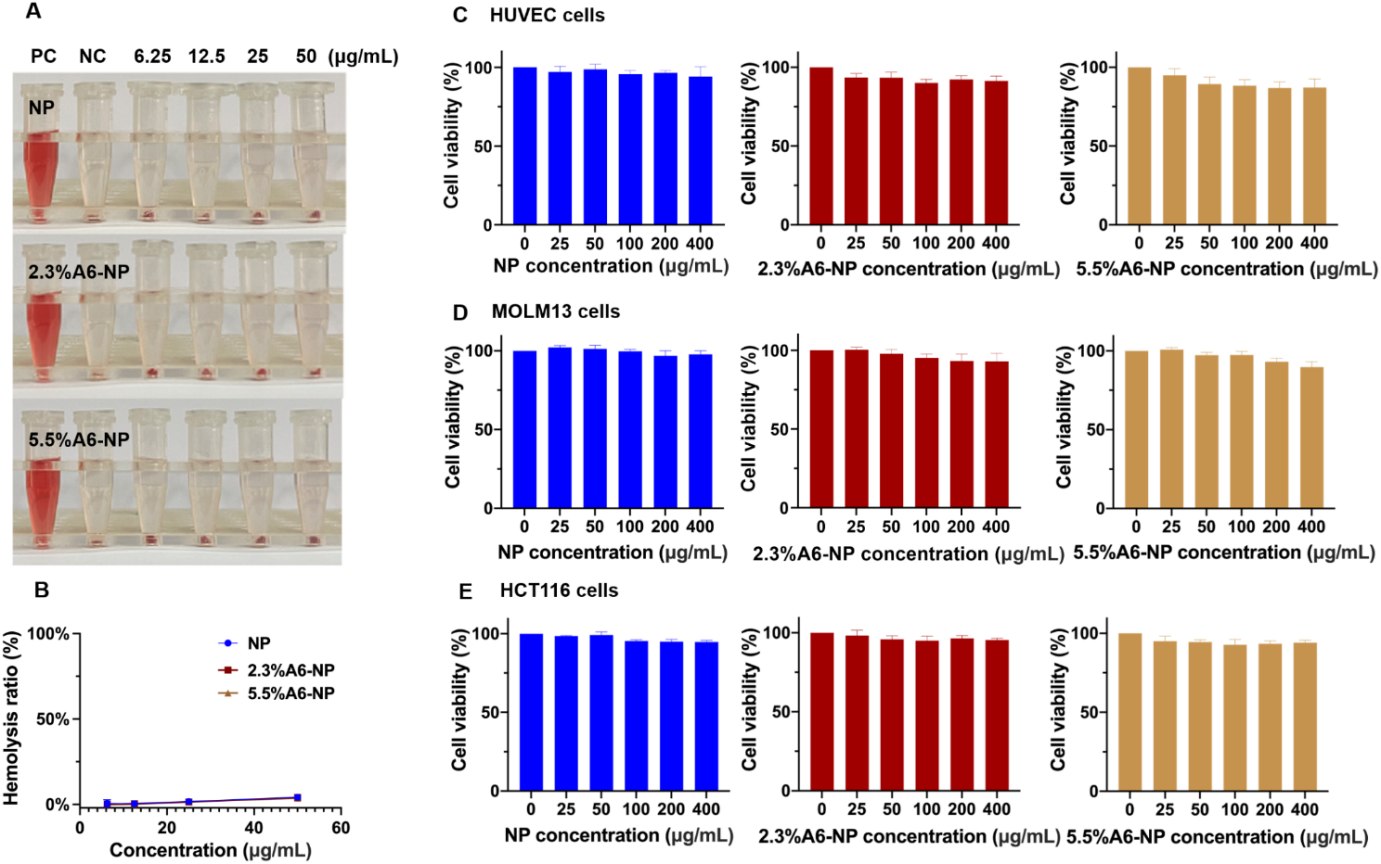
Biocompatibility of A6-NP. (**A**) Hemolysis test of fabricated nanoparticles. (**B**) Hemolysis ratio of fabricated nanoparticles. (**C**) Cell viabilities of blank nanoparticles in HUVECs (*n* = 3). (**D**) Cell viabilities of blank nanoparticles in MOLM13 cells (*n* = 3). (**E**) Cell viabilities of blank nanoparticles in HCT116 cells (*n* = 3)

For in vivo therapeutic applications, nanoparticles should be nontoxic and biocompatible. To further study the biocompatibility of nanoparticle treatment, we examined the hemolytic properties of fabricated nanoparticles. As illustrated in Fig. 4A and B, in the hemolysis assay, the hemolysis rate of NP was less than 5% (at concentrations of 6.25, 12.5, 25 and 50 µg/mL). In addition, hemolysis rates of functionalized nanoparticles (2.3%A6-NP and 5.5%A6-NP) were both less than 5% and the hemolysis rate was almost the same as that of NP at the same incremental concentrations. These results demonstrated that NP, 2.3%A6-NP and 5.5%A6-NP have great hemocompatibility and that A6 peptide modification with SMCC does not affect the hemocompatibility of human serum albumin.

The cytotoxicity of blank-NP, blank-2.3%A6-NP and blank-5.5%A6-NP was investigated in HUVECs, MOLM13 cells and HCT116 cells. HUVECs, as the main component of the inner lining of major blood vessels, are likely to be the first active components that nanoparticles intravenously encounter [42]. The cytotoxicity in HUVECs was first estimated, as displayed in Fig. 4C. As high as 400 µg/mL of blank-NP had well safety with cell viability higher than 95% after 48 h of incubation. However, compared with that in the blank-NP group, the modification of A6 peptide slightly affected the activity of HUVECs, and the higher the degree of modification of A6 peptide was, the greater the cytotoxicity, with 86.8% cell viability at 400 µg/mL in the blank-5.5%A6-NP group. The result can be explained by that A6 peptide has anti-angiogenic, anti-invasive and antimigratory properties [43, 44].

For leukemia MOLM13 cells, blank-NP, blank-2.3%A6-NP and blank-5.5%A6-NP showed great safety, as all cell viabilities were greater than 90%. Besides, there was no significant difference in cell viability between the blank-NP group (97.8 ± 2.2%) and the blank-2.3%A6-NP group (92.9 ± 5.5%) at the highest concentration of 400 µg/mL. However, the viability of cells treated with 5.5%A6-NP (90.7 ± 3.9%) was remarkably lower at 400 µg/mL than that of cells treated with blank-NP. In solid colon cancer HCT116 cells, all fabricated nanoparticles exhibited well safety, as all the cell viabilities were greater than 90%, and there were no significant differences among NP, 2.3%A6-NP and 5.5%A6-NP groups at any concentration (Fig. 4D and E). It has been reported that A6 has antiangiogenic, anti-invasive and antimigratory, but is not anti-proliferative [43, 44]. Therefore, from the above results, it is not surprising that the greater the amount of A6 conjugated on the surface of nanoparticles, the better.

### In vitro antileukemia activities, antitumor activities, and apoptosis assays

**Fig. 5:**
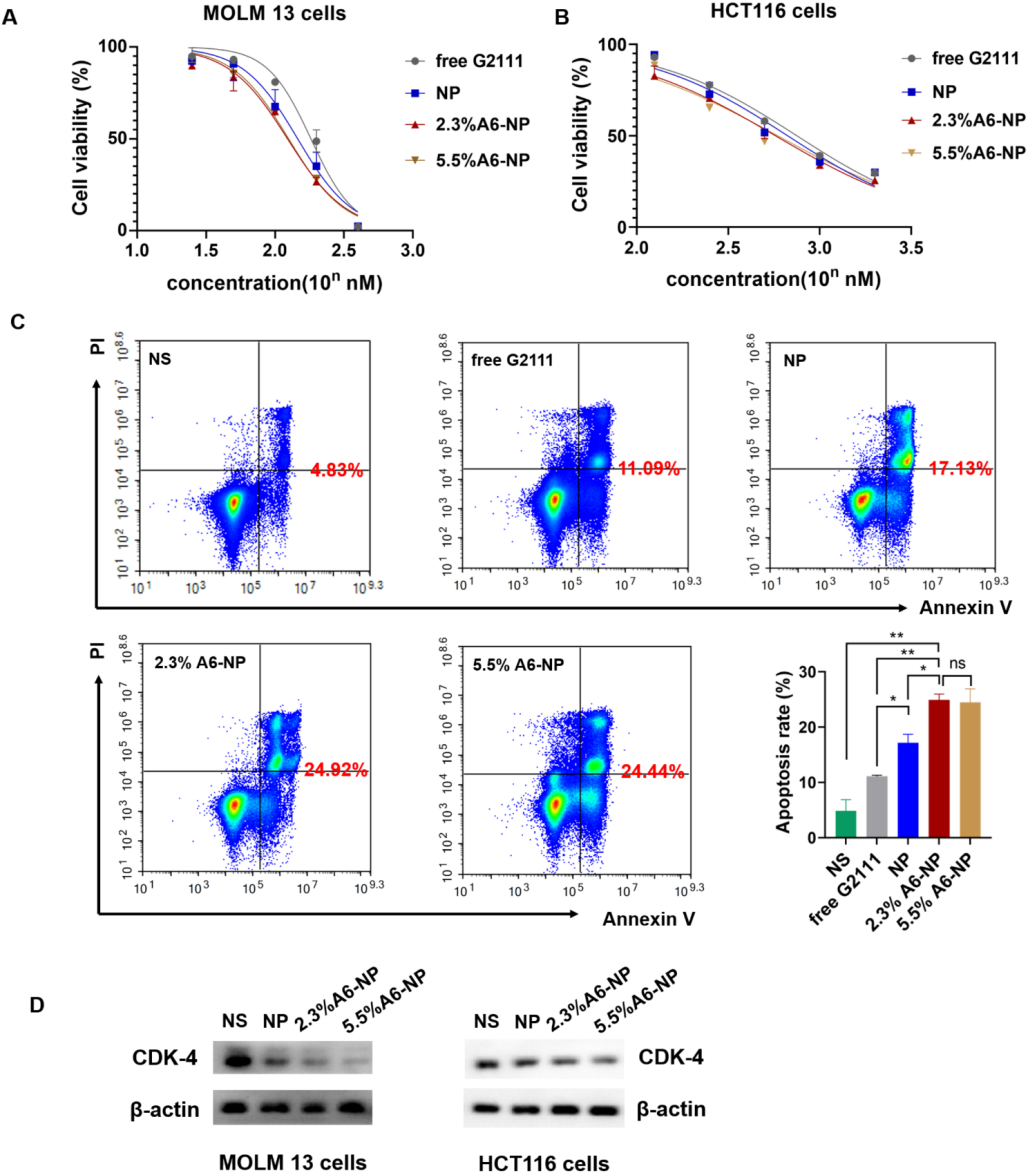
In vitro anticancer activity of fabricated nanoparticles. (**A**) Cell viability of MOLM-13 cells treated with different formulations for 48 h (*n* = 3). (**B**) Cell viability of HCT116 cells treated with different formulations for 48 h (*n* = 3). (**C**) Apoptotic assays of MOLM-13 cells treated with different formulations (*n* = 3). (**D**) Western blot assay of CDK-4 in MOLM13 and HCT116 cells treated with NP, 2.3%A6-NP and 5.5%A6-NP, respectively.   ^*^p < 0.05, ^**^p < 0.01, ^***^p < 0.001

We further determined in vitro antileukemic efficacies of free G2111, NP, 2.3%A6-NP and 5.5%A6-NP in MOLM-13 cells, and in vitro antitumor efficacies in HCT116 cells. As detailed in Fig. 5A, NP, 2.3%A6-NP and 5.5%A6-NP all had higher cytotoxicity than free G2111 (IC50 = 139.0 nM), indicating that G2111 could be efficiently released from nanoparticles in cells. Compared with that of NP (IC_50_ = 111.6 nM), the antileukemic efficacy of the two A6-NP formulations was greater. The CD44 receptor-mediated endocytosis of A6-based nanoparticles might contribute to the enhanced cytotoxicity of A6-NP, and these results were in accordance with the results of the cellular uptake assay. It has been reported that the density of ligand on nanoparticles plays a significant role in their targeting ability [45]. Interestingly, with increasing A6 density on albumin-based nanoparticles, the antitumor efficacy did not increase and IC_50_ of 2.3%A6-NP and 5.5%A6-NP was 100.9 nM and 103.0 nM, respectively (Fig. 4A). For HCT116 cells, the results were similar to those for MOLM13 cells. The IC_50_ of 2.3%A6-NP (IC_50_ = 591.2 nM) was significantly lower than that of NP (IC_50_ = 713.7 nM), demonstrating that A6 peptide augmented the antitumor activity of NP via CD44 mediated internalization. However, there was little difference in the IC_50_ between 2.3%A6-NP and 5.5%A6-NP (Fig. 5B). These results indicated that G2111 could be efficiently released from A6-NP and NP in both MOLM-13 and HCT116 cells, and that anticancer activity of G2111 could be improved by A6-mediated endocytosis.

Hsp90 acts as chaperone by folding its client proteins to mature and maintain their active conformations. More than half of Hsp90 client proteins are oncological proteins, such as CDK4, HER2, EGFR, etc. [46, 47]. Therefore, inhibition of Hsp90 could negatively affect the expression level of client proteins. So, we evaluated the effect of nano-formulations on the expression of Hsp90 client protein CDK4 which is associated with cell proliferation. As exhibited in Fig. 5D, all formulations significantly downregulate the expression of CDK4 in MOLM13 and HCT116 cells with a A6 peptide modification dependent manner, A6-NP groups more remarkable than NP groups. Hence, A6-NP may effectively inhibit the expression of CDK4 which can prevent cell proliferation through cell-cycle progression arrest.

We further determined cell apoptosis rates of MOLM13 cells treated with free G2111, NP, 2.3%A6-NP and 5.5%A6-NP. The flow cytometry data (Fig. 5C) exhibited that the percentage of apoptosis in 2.3%A6-NP group was 24.92%, which was remarkably greater than that of NP group (*p* < 0.05). Although the level of A6 peptide in 5.5%A6-NP group was higher than that in the 2.3%A6-NP group, there was no obvious difference in cell apoptosis. These results revealed that A6 peptide active-CD44 targeting played a vital role in antitumor efficacy. Notably, an increase in the amount of A6 in nanoparticles is unlikely to lead to a more powerful antitumor suppression effect when the content of A6 peptide reaches a certain level. To further verify the effect of the content of A6 peptide on the in vivo anticancer activities, both 2.3%A6-NP and 5.5%A6-NP were used for subsequent studies.

### Antileukemia activity in orthotopic AML mice

**Fig. 6:**
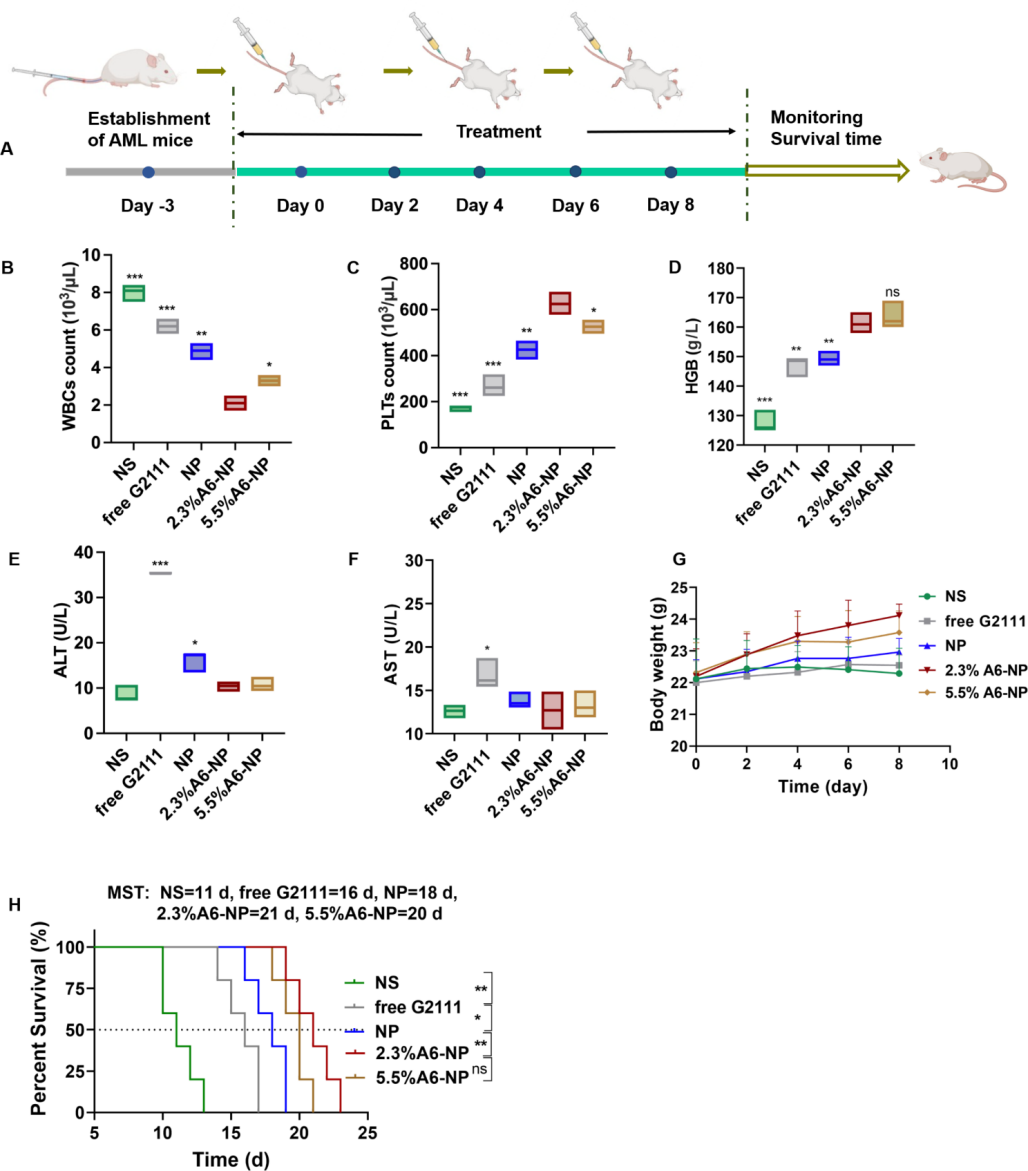
(**A**) Overview of the experimental design of in vivo antileukemia activity (*n* = 5). (**B**) WBC counts, (**C**) PLT counts, (**D**) HGB level of peripheral blood collected from the eye socket of the AML mice on day 9. (**E**) ALT level in serum. (**F**) AST level in serum. (**G**) Body weight changes of mice. (**H**) Survival curves of mice

The in vivo antileukemia efficacy of A6-NP was conducted in orthotopic leukemia mice. These formulations were administered every other day for 5 times, with free G2111 as a comparison (Fig. 6A). Mice body weight monitoring was performed during the treatment period. As displayed in Fig. 6G, the mice treated with 2.3%A6-NP and 5.5%A6-NP displayed obvious increasing trends in body weight, revealing that 2.3%A6-NP and 5.5%A6-NP are efficient and safe for the treatment of AML mice. Notably, compared to that of 5.5%A6-NP group, the body weight of 2.3%A6-NP-treated group increased distinctly, indicating that 2.3%A6-NP exerted a better antileukemic effect with lower systemic toxicity. Peripheral blood routine analysis presented that WBC counts in free G2111 and NP groups were obviously decreased after treatment (Fig. 6B). The number of WBCs in mice treated with 2.3%A6-NP showed a significant decreasing trend in comparison with that in NP group (*p* < 0.01). In addition, in the two A6-conjugated nanoparticle-treated groups, 2.3%A6-NP exhibited the better ability to reduce WBC counts than 5.5%A6-NP (*p* < 0.05). Moreover, higher levels of PLTs and HGB (as shown in Fig. 6C and D) were observed in 2.3%A6-NP group than in the NP group (both *p* < 0.01 for PLTs and HGB). For the different A6 peptide degree-modified groups, PLTs in 2.3%A6-NP group were markedly higher than those in the 5.5%A6-NP group (*p* < 0.05), while there was no obvious difference in HGB between the two groups. These findings indicated that 2.3%A6-NP greatly relieved the burden of leukemia and further enhanced the safety of Hsp90 inhibitors in the hematologic system by grace of the CD44 targeting via appropriate A6 peptide modification (2.3%). However, it is not necessarily better to have a large amount of A6 on nanoparticles. One reason is that a large amount of A6 probably provides more opportunities for nanoparticles to enter normal cells with low CD44 expression. Another reason is that A6 itself also has other physiological activities, such as antiangiogenic, anti-invasive and antimigratory effects [43, 44], which may have an impact on antileukemia efficacy and safety. In our study, 2.3% A6-NP exhibited improved antileukemia efficacy. Further increasing the amount of A6 (5.5%) did not improve the antileukemia effect, and it likely increased the risk of adverse events. According to the results of the in vitro cytotoxicity evaluation on HUVECs (Fig. 4 (C)), the greater the degree of modification of the A6 peptide was, the greater the cytotoxicity, with 86.8% cell viability at 400 µg/mL in the blank-5.5%A6-NP group.

Hepatotoxicity is the major adverse reaction of Hsp90 inhibitor geldamycin and its derivatives, 17AAG has significantly improved liver tolerance in clinical trials [48, 49]. However, G2111 we synthesized, showed higher activity than 17AAG but lower liver toxicity [38]. To further investigate the hepatotoxicity of fabricated nanoparticles, the serum alanine transaminase (ALT) and aspartate transaminase (AST) levels were measured after treatment at day 9. As shown in Fig. 6E and F, the levels of ALT and AST in free G2111-treated mice were obviously increased in comparison to the NS group, indicating that the solvent (Ethanol: Cremphor EL = 1:1) for free G2111 caused a certain degree of hepatotoxicity. Compared with those in the NP group, 2.3% A6-NP and 5.5% A6-NP significantly reduced the ALT level, and there was no significant difference between the A6-NP group and the saline group. As for AST levels, there was not remarkably different among NS, NP, 2.3% A6-NP and 5.5% A6-NP groups. Since ALT is found mostly in the cytosol of the liver and at low levels elsewhere, and compared to AST, which is present in many cell types, ALT is more specific for hepatic damage [50]. Hence, from the above results, A6 peptide-conjugated albumin nanoparticles could further avoid hepatotoxicity of G2111. It is probable that A6 as target heads leads to increased accumulation of nanoparticles in organs such as bone marrow cavities where CD44-positive leukemia cells are abundant, thereby reducing the aggregation of nanoparticles in other organs such as the liver and further reducing the hepatotoxicity of G2111.

**Fig. 7:**
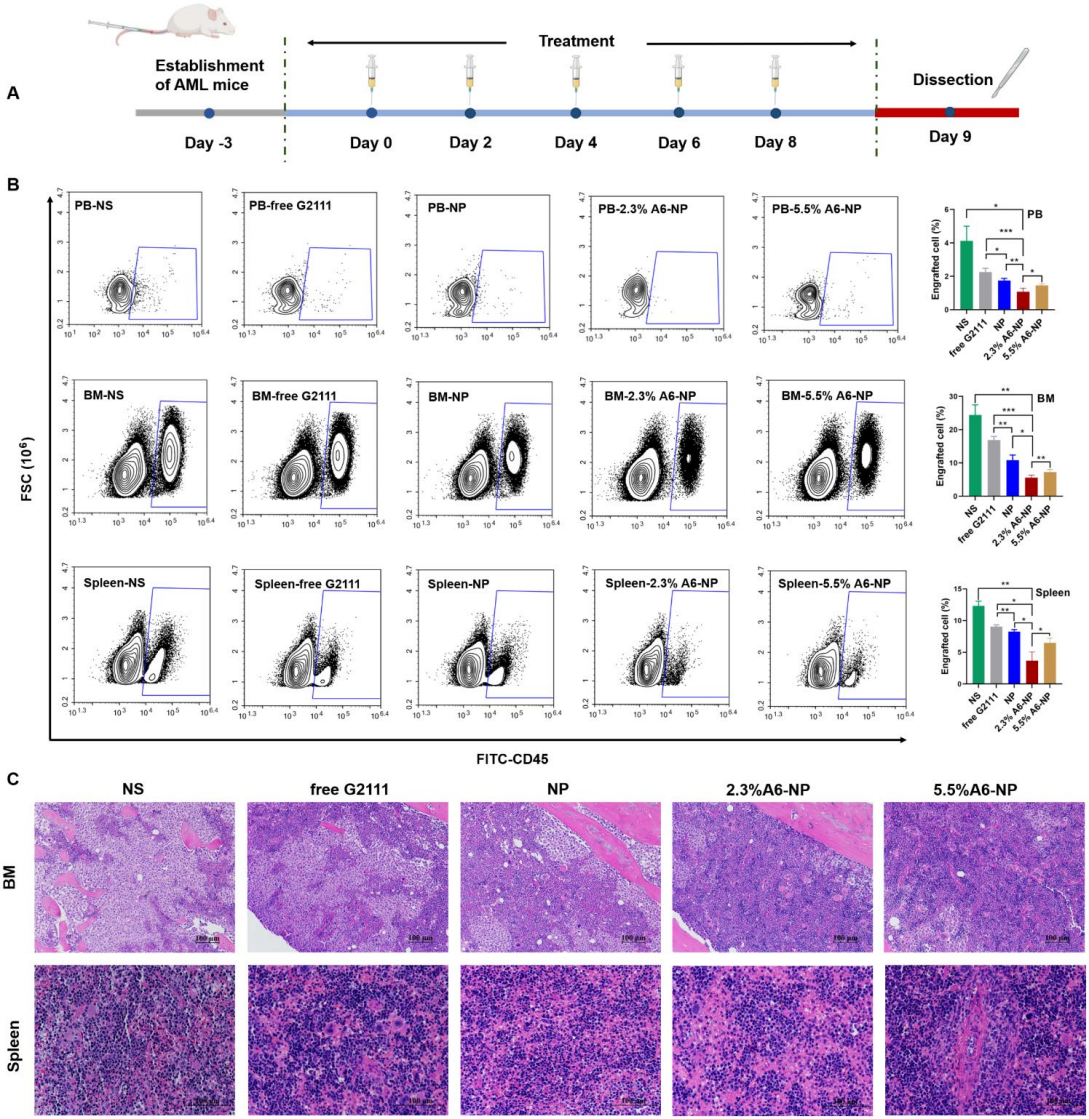
(**A**) In vivo experimental scheme of leukemia cells infiltrated. (**B**) Infiltration analysis of leukemia cells in peripheral blood (PB), bone marrow (BM) and spleen determined by flow cytometry (*n* = 3). ^*^p < 0.05, ^**^p < 0.01, ^***^p < 0.001. (**C**) Representative views of H&E-stained BM and spleen sections, scale bar 100 μm

**Fig. 8:**
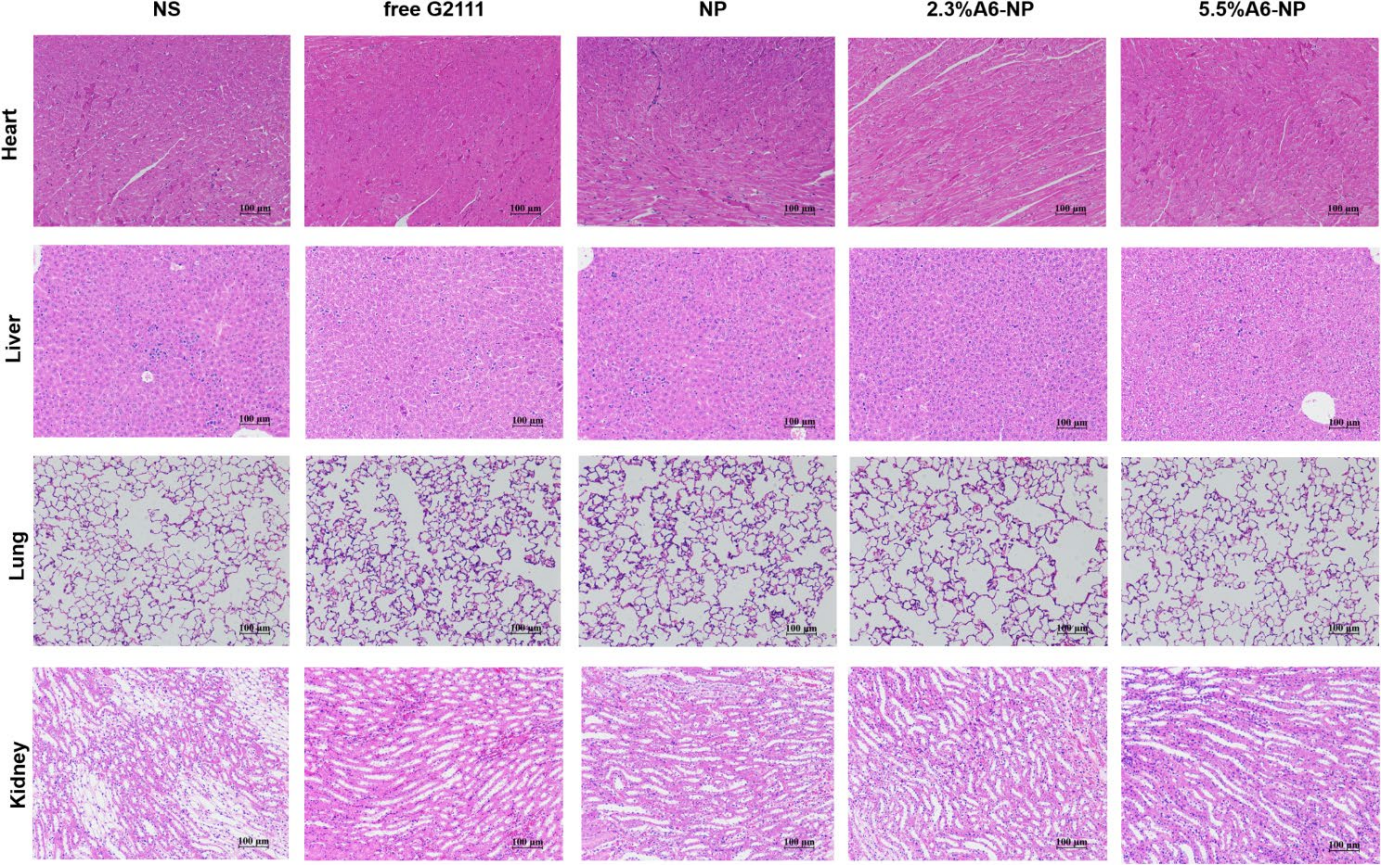
Histological evaluation for tissue damage of the major organs. Representative views for H&E of heart, liver, lung and kidney slices, scale bar 100 μm

From the survival curves in Fig. 6H, it was shown that the median survival time (MST) for the NS group was only 11 days, demonstrating the seriously aggressive nature of acute myeloid leukemia. These survival curves displayed that NP improved the survival of AML mice compared to the free G2111 group, with MST increased from 16 days to 18 days. Notably, the MST of 2.3%A6-NP group was 21 days, showing an excellent effect on prolonging survival. As for the therapeutic efficiency of 5.5%A6-NP group, there was no remarkable differences in MST (in comparison with 2.3%A6-NP group). The massive invasion of leukemia cells can lead to impaired normal hematopoiesis. As amount of leukemia cells increases, the hematopoietic function of the bone marrow gradually depletes, causing animal death. Compared with G2111 and NP, 2.3% A6-NP has well targeted capacity, better safety and antileukemia efficacy in the bone marrow, peripheral blood and spleen (as exhibited in detail in Figs. 7 and 8), resulting in a longer survival period.

To further evaluate in vivo leukemia cell infiltration after treatment with A6-NP, the schedule of administration was exhibited in Fig. 7A and all mice were dissected on day 9. The leukemia cells that infiltrated the peripheral blood (PB), spleen and bone marrow (BM) were harvested and assessed by flow cytometry. Figure 7B shows that NS group presented tremendous invasion of leukemia cells for PB, BM and spleen. Compared with those in the free G2111 (*p* < 0.001) and NP (*p* < 0.01) groups, the proportion of leukemia cells in the PB in the 2.3%A6-NP group was remarkably decreased. Leukemia invasion in the spleen and BM was consistent with that in the PB group. Besides, the reduction in leukemia invasion in the PB, BM and spleen of mice treated with 2.3%A6-NP was significantly lower than that of 5.5%A6-NP (*p* < 0.05, *p* < 0.01 and *p* < 0.05, respectively). Additionally, H&E staining of the engrafted leukemia cells revealed that numerous leukemia cells were located in the BM of the NS group, as shown in Fig. 7C, while 2.3%A6-NP and 5.5%A6-NP remarkably decreased the invasion of leukemia cells, and abundant hematopoietic cells were observed in the BM of the 2.3%A6-NP and 5.5%A6-NP groups. In the spleen, infiltration was considerably reduced in the 2.3%A6-NP and 5.5%A6-NP groups, similar to that of BM. Furthermore, histopathological changes in heart, liver, lung and kidney were analyzed using H&E staining (Fig. 8). Histological evaluation showed no obvious signs of histopathological changes due to tissue damage to the major organs, confirming the safety and utility of this nanoparticle system for the delivery of G2111 to AML. Taken together, these results demonstrated that CD44-targeting A6 peptide functionalized HSA-based nanoparticles could significantly improve the antileukemia effect of AML both in vitro and in vivo, and 2.3% modified nanoparticles exhibited the greatest anti-leukemia effect.

### Targeted accumulation and antitumor efficacy of A6-NP in solid tumor HCT116 colon tumor-bearing mice

**Fig. 9:**
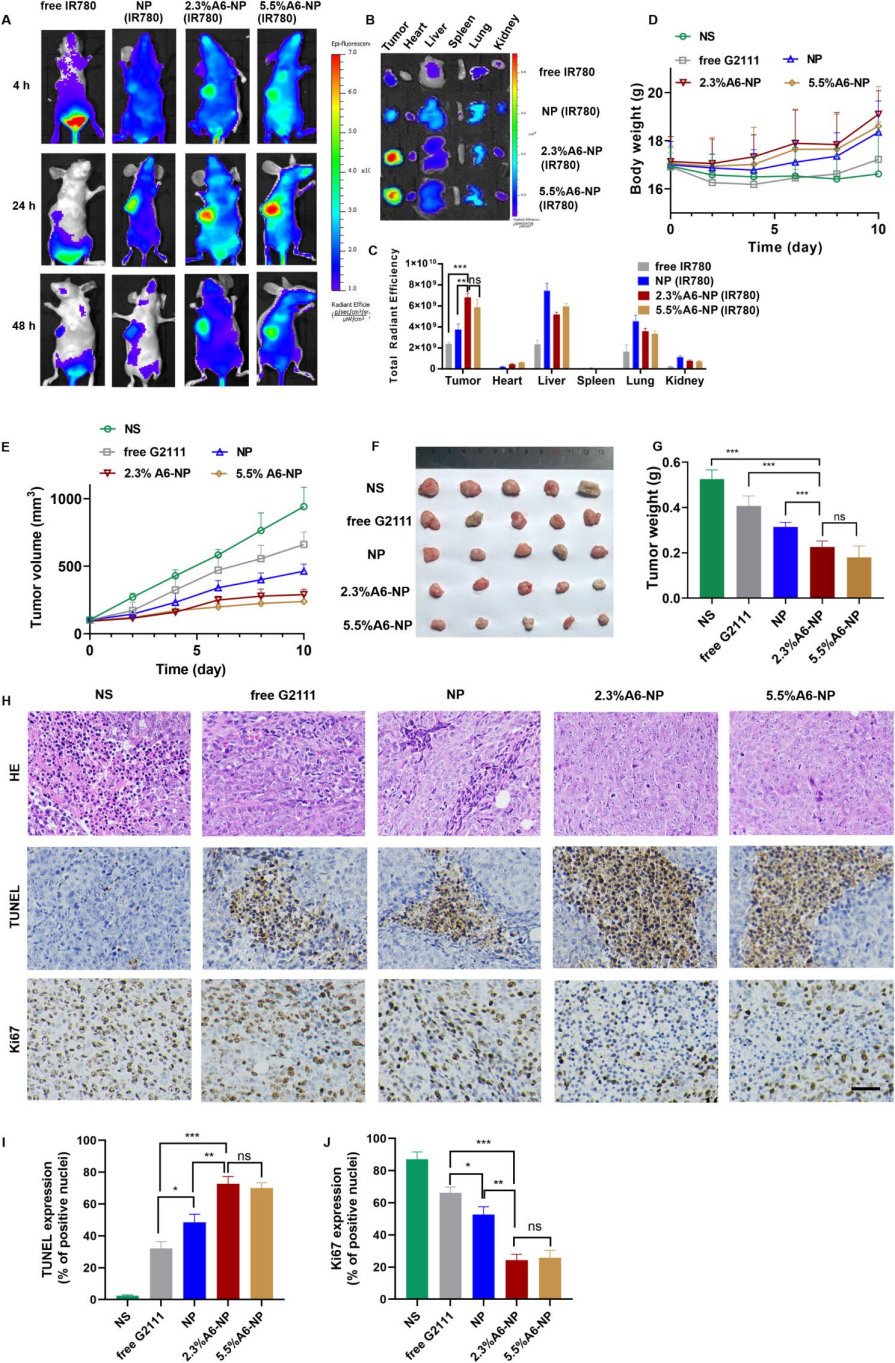
(**A**) In vivo fluorescence images of HCT116 tumors at different time points after i.v. injection of IR780-loaded nanoparticles. (**B**) Ex vivo images of main organs and tumors from mice treated with IR780-loaded nanoparticles. (**C**) Semi-quantitative analysis of fluorescence intensity of tumor and main organs *ex vivo.* (**D**) Body weight changes of mice. (**E**) Tumor volume of each group. (**F**) Tumor images of each group. (**G**) Tumor weight of each group (**H**) H&E, TUNEL and Ki67 staining of tumor sections, scale bar 50 μm. Quantitative results of TUNEL (**I**) and Ki67 (**J**).

HCT116 colon tumor-bearing xenograft mice models were established as solid tumor animal models to assess the ability to target to CD44^+^ solid tumors and the antitumor efficacy of A6-NP in vivo. We investigated the in vivo tumor targeting ability of A6-NP by injecting IR780-labeled nanoparticles (A6-NP(IR780)) into HCT116 colon tumor-bearing mice through the tail vein. The distribution and tumor accumulation were observed through fluorescence imaging at 4 h, 24 h and 48 h after intravenous injection. As shown in Fig. 9(A), strong fluorescence signals of A6-NP(IR780) were observed in the tumors and remained for up to 48 h, demonstrating that A6-NP could efficiently improve tumor accumulation and retention. Compared to free IR780 and IR780-loaded NP, 2.3%A6-NP and 5.5%A6-NP exhibited an evident tumor distribution. Human serum albumin may endow A6-NP with the characteristic of long circulation. Tumor cells actively take up a large amount of protein to metabolize and replenish energy due to their high proliferation, which can induce the accumulation of A6-NP in tumors. Moreover, A6 peptides reinforce the nanomedicine’s tumor-targeting capacity and can further increase and prolong tumor accumulation. To further verify essential role of A6 peptide in reinforcing the nanomedicine’s tumor-targeting capacity, tumor-bearing mice were sacrificed at 24 h and the main organs and tumor tissues were collected and the fluorescence intensity was quantified (Fig. 9B and C). As shown in Fig. 9C, NP significantly increased the fluorescence intensity in tumor tissues compared with that in the solution group, probably because NPs are mainly composed of proteins, and tumor cells actively take up a large amount of protein to replenish energy for high proliferation. Compared with those in the NP group, the 2.3%A6-NP and 5.5%A6-NP groups exhibited stronger fluorescence in tumors and weaker fluorescence in other main organs, indicating that A6 peptides improved tumor-targeting capacity for accumulation of nanoparticles in tumors. These in vivo and ex vivo fluorescence results confirmed that the active targeting capacity of A6 peptide could remarkably improve the colon-tumor targeting ability of our nanoparticles.

The antitumor effect of A6-NP on solid tumors in vivo was evaluated in HCT116 colon tumor-bearing mice after treatment with the formulations every 2 days for 5 times. Changes in tumor volume and body weight were monitored every two days during the administration. As shown in Fig. 9(E), free G2111 slightly suppressed the growth of HCT116 colon tumors. NP mediated more effective tumor inhibition than free G2111. Notably, A6-NP led to tumor shrinkage during the treatment period, and 2.3%A6-NP and 5.5%A6-NP significantly inhibited tumor growth compared with the nontargeted NP. However, as shown in Fig. 9(F-G), the antitumor activities of 2.3%A6-NP and 5.5%A6-NP were not remarkably different. It has been reported that A6 has antiangiogenic, anti-invasive and antimigratory activities [42, 43], which probably suppress solid tumor HCT116 colon tumor growth. However, in our study, a greater amount of the A6 peptide did not induce better antitumor effects, and there was no significant difference between 2.3%A6-NP and 5.5%A6-NP, indicating that the A6 peptide mainly acts as a target head in A6-NP. A6 peptides improved the nanomedicine’s tumor-targeting capacity and drug accumulation in tumors; thus, A6-NP exhibited better antitumor efficacy than free G2111 and nontargeted NP.

**Fig. 10:**
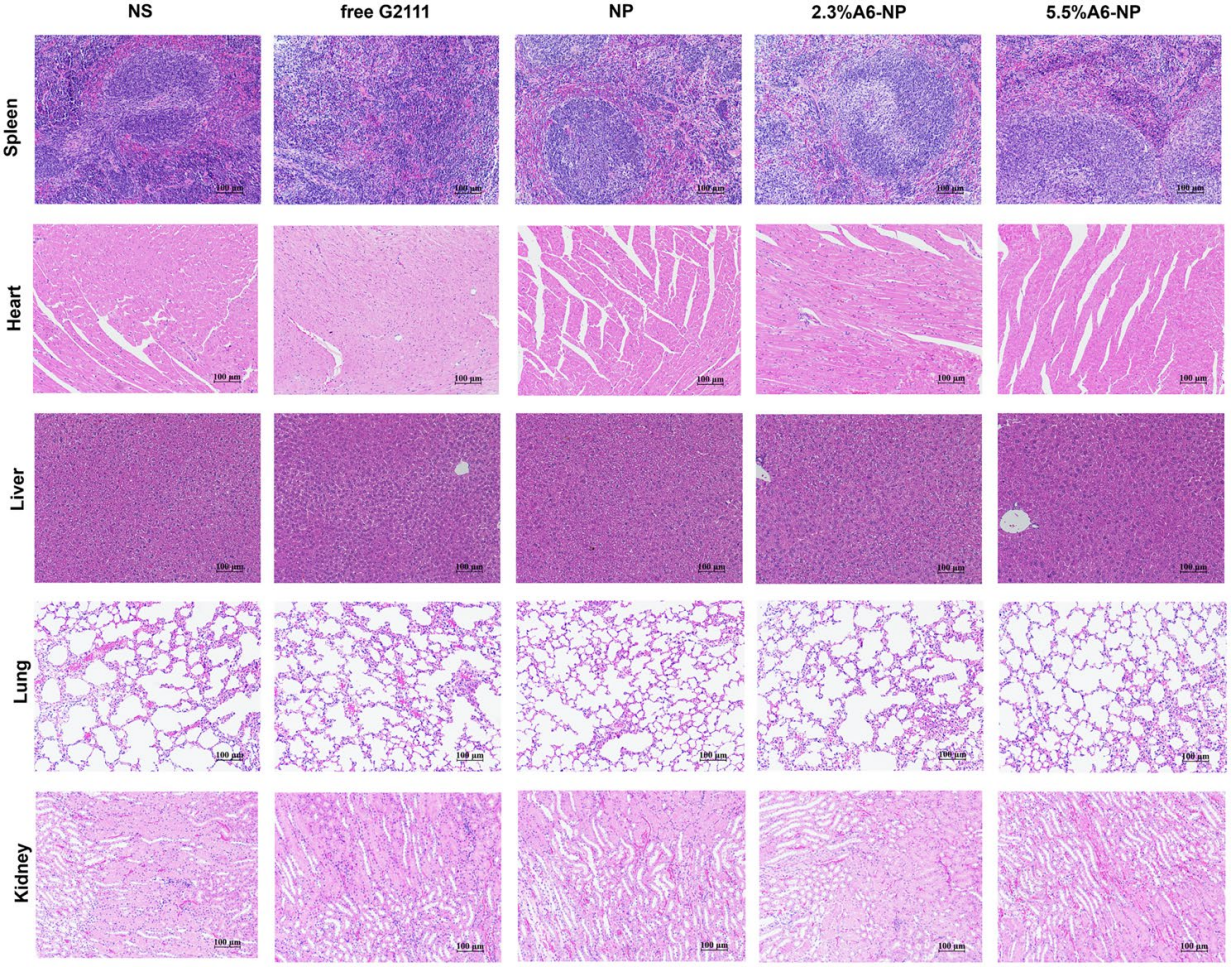
Representative views of H&E-stained spleen, heart, liver, lung and kidney sections, scale bar 100 μm

Besides, the histological analyses of tumors using H&E and TUNEL staining revealed extensive necrosis and apoptosis of tumor cells in the 2.3%A6-NP and 5.5%A6-NP groups (Fig. 9H). Figure 9H and I show low levels of apoptosis in the saline and free G2111 groups but markedly increased levels in the three NP groups. The 2.3%A6-NP and 5.5%A6-NP groups had greater levels of apoptosis than did the NP group (*P* < 0.01). However, there was no significant difference between 2.3%A6-NP and 5.5%A6-NP groups. Ki67 is a marker that is associated with tumor cell proliferation and was highly expressed in the NS group (Fig. 9J). NP and A6-NP significantly reduced Ki67 expression; moreover, A6-NP reduced Ki67 expression down to 24%. While there was no remarkably difference between 2.3%A6-NP and 5.5%A6-NP. These results revealed that A6-NP had better antitumor activities compared with free G2111 and NP. The above results were consistent with the tumor suppression data. During the treatment, the body weights of mice were monitored, as shown in Fig. 9D. 2.3%A6-NP and 5.5%A6-NP groups had an upward trend in body weight, indicating low systemic toxicity with improved antitumor activity. In comparison, free G2111 group showed a slight weight gain during the treatment, while the NS group showed a slight fluctuation. H&E staining results also displayed no obvious histological changes in major organs of mice (Fig. 10). These results demonstrated that the A6-NP had good biocompatibility and biosafety in vivo.

## Conclusions

In this study, we revealed that CD44-targeted A6 peptide functionalized biomimetic nanoparticles possesses remarkable targetability and anticancer activities toward orthotopic human acute myeloid leukemia xenografts and heterotopic human colon tumor xenografts in vivo, lending to significant survival benefits and avoid hepatotoxicity compared to unmodified nanoparticles as well as free HSP90 inhibitor. Moreover, A6-NP, while holding desired merits including well biocompatibility, no excipients, excellent stability, active-targetability and good safety, is easy and green to fabricate, reinforcing it a high potential for clinical translation. Meanwhile, our fabricated A6 peptide functionalized nanoparticles with CD44 and Hsp90 targets display a broad anti-cancer spectrum both in CD44 positive hematological malignancies and solid tumors, which are rarely reported in one nanomedicine, and it is a promising strategy to therapy hematological malignancies and solid tumors.

## Data Availability

All data generated or analyzed during this study are included in this manuscript.
