# Correction: Targeted delivery of HSP90 inhibitors for efficient therapy of CD44-positive acute myeloid leukemia and solid tumor-colon cancer

**DOI:** 10.1186/s12951-026-04333-1

**Published:** 2026-04-27

**Authors:** Lejiao Jia, Huatian Yang, Yue Liu, Ying Zhou, Guosheng Li, Qian Zhou, Yan Xu, Zhiping Huang, Feng Ye, Jingjing Ye, Anchang Liu, Chunyan Ji

**Affiliations:** 1 Department of Pharmacy, Cheeloo College of Medicine, Qilu Hospital, Shandong University, Jinan, 250012 Shandong China; 2 Department of Pharmaceutics, School of Pharmaceutical Sciences, Cheeloo College of Medicine, Shandong University, Jinan, 250012 Shandong China; 3 Department of Pharmacy, Affiliated Hospital of Shandong University of Traditional Chinese Medicine (TCM), Jinan, 250014 Shandong China; 4 Department of Hematology, Cheeloo College of Medicine, Qilu Hospital, Shandong University, Jinan, 250012 Shandong China; 5 Key Laboratory of Chemical Biology of Ministry of Education, Schoolof Pharmaceutical Sciences, Cheeloo College of Medicine, Shandong University, Jinan, 250012 Shandong China

**Correction: Journal of Nanobiotechnology (2024) 22:198**

10.1186/s12951-024-02460-1

Upon careful re-examination of raw data and figure source files, the authors identified that the duplication alert resulted from an inadvertent error during figure assembly process (Fig. 10 (Lung)).

The authors confirmed that this error occurred during figure assembly process does not compromise the integrity of the data, results, or conclusions of the research. Besides, H&E staining results from the free G2111, NP, 2.3% A6-NP, and 5.5% A6-NP groups in Fig. 10 displayed no obvious histological abnormalities in Lungs.

In this article Fig 10 appeared incorrectly and has now been corrected in the original publication. For completeness and transparency, the old incorrect and correct versions are displayed below. The original article has been corrected.

Incorrect Fig. 10



Correct Fig. 10

**Fig. 10:**
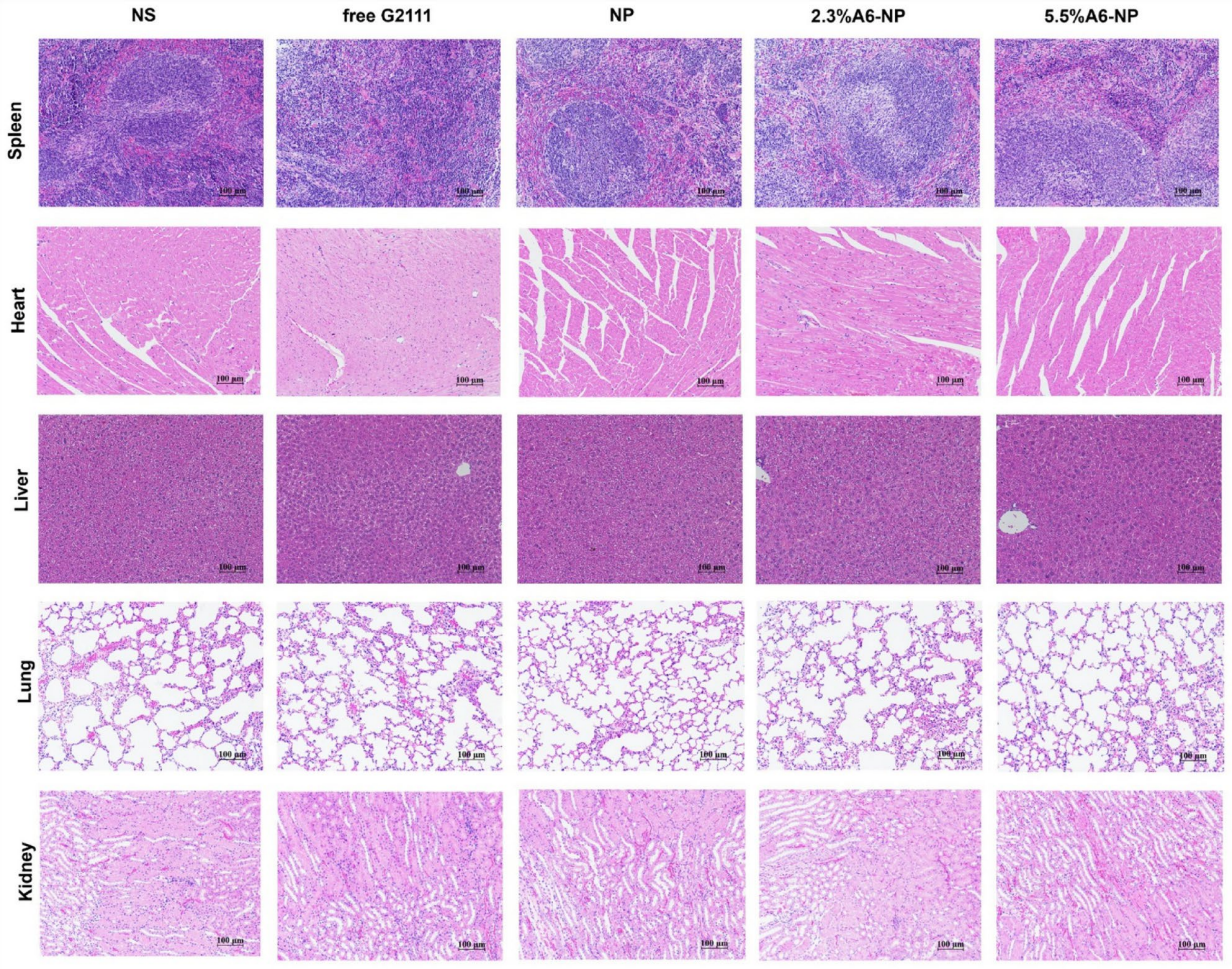
Representative views of H&E stained spleen, heart, liver, lung and kidney sections, scale bar 100 μm

